# Supplementary material for: Dynamic transcriptomic profiles of zebrafish gills in response to zinc supplementation
Source: BMC Genomics. 2010 Oct 11;11:553. doi: 10.1186/1471-2164-11-553 (PMC3091702; doi:10.1186/1471-2164-11-553)
Supplement: Additional file 2 — Interactive Direct Interaction Network representing the molecular interactions between zinc, copper, iron, calcium and proteins encoded by transcripts changed by zinc supplementation. Mini web-site containing index.html and hyperlinked pages in subdirectory describing a Direct Interaction Network automatically generated based on curated interactions contained within the proprietary PathwayArchitect database. Ovals represent proteins and the circles symbolize metal ions. Objects are coloured by their abundance in zebrafish at the time-point they were significantly different from the control is a scale from -4 fold (dark green) to +4 fold (dark red). Where significant differences were found at more than one time-point, the colour overlay shows expression at the first instance. Dark blue squares denote 'binding', and light blue squares 'expression'; green squares stand for 'regulation', green diamonds for 'metabolism', and green circles for 'promoter binding'. Arrow heads indicate directionality of the interaction where annotated. All nodes and edges can be further interrogated by selecting the relative area of the image. [file 1471-2164-11-553-S2.zip › PathwayArchitect Zn xs DIN/581230.html]

# REGULATION:

|  |  |
| --- | --- |
| Type | REGULATION |
| Effect | Positive |


---

|  |  |
| --- | --- |
| Score | 0 |


---

|  |  |
| --- | --- |
| Reference Count | 4 |


---

|  |  |
| --- | --- |
| Mechanism | Unknown |


---

|  |  |
| --- | --- |
| Reference:0 || Sentence | "A similar pattern of subcellular redistribution and colocalization of BAX with BID and BAX with VDAC-1 suggests that BAX (in association with BID) controls the function of VDAC-1 and its permeability for apoptogenic factors released from mitochondria of COLO 205 cells stimulated to apoptosis with NIM." |
| PMID | 12439336 |
| Year | 2002 |
| Species | Human |
| Journal | Anticancer Drugs |
| RefScore | 1 |
| Source | PArchNLP |
  |
|  |  |


---

|  |  |
| --- | --- |
 Reference:1 || Sentence | "Here, we investigated the role of ANT and VDAC in the changes of isolated mitochondria triggered by Bax and by chemicals that induce permeability transition (PT)." |
| PMID | 10980606 |
| Year | 2000 |
| Species | Rat |
| Journal | Oncogene |
| RefScore | 2 |
| Source | PArchNLP |
  ||  |  |


---

|  |  |
| --- | --- |
 Reference:2 || Sentence | "We conclude that Bax does not induce cytochrome c release by acting on VDAC." |
| PMID | 14729675 |
| Year | 2004 |
| Species | Rat |
| Journal | J Biol Chem |
| RefScore | 2 |
| Source | PArchNLP |
  ||  |  |


---

|  |  |
| --- | --- |
 Reference:3 || Sentence | "Furthermore, BH4 oligopeptides of Bcl-2 and Bcl-x(L), but not mutant peptides, were able to inhibit both VDAC activity on liposomes even in the presence of Bax and apoptotic Deltapsi loss in isolated mitochondria." |
| PMID | 10737788 |
| Year | 2000 |
| Species | Human |
| Journal | Proc Natl Acad Sci U S A |
| RefScore | 2 |
| Source | PArchNLP |
  |


---

|  |  |
| --- | --- |
